# A Century of Rebuilding at Leeds

**Published:** 1917-03-24

**Authors:** 


### A CHAPTER IN HOSPITAL HISTORY.

### A Century of Rebuilding at Leeds.

Hospital histories are always welcome, and the anonymous author of "A Historical Sketch of the General Infirmary, Leeds," deserves thanks for his work in tracing the lines of the hospital's development from its foundation in 1767 to the present day. William Hey, F.R.S., one of John Hunter's pupils, whose statue is still to be seen, was one of the active spirits at this early date, when the City of Leeds, the centre of the woollen trade, had no hospital for its 17,000 inhabitants. He died in 1819, in the same year as Mr. Mathew Talbot, a man of learning, the first secretary, who held the post for thirty-three years. When we recall that at this date there was no drainage, no street lighting, and a poor and probably tainted water supply, we realise how everything which we regard as the normal sign of civilised town life is but the creation of the last seventy years. The inn was then the club, the meeting-place, and the hall of assembly, and at a meeting at one of these pleasant taverns it was decided in the summer of 1767 to appoint Mrs. Mary Turner, of "fifty years and upwards," matron of the new infirmary in Kirkgate, at a salary of £10, with Nurse Atkinson, as her staff, at £5 per annum.

### The First New Building.

In a year's time the foundation-stone of a new building was laid on the site of the present General Post Office, where patients were received on March 1, 1771. In 1778 John Howard, the philanthropist, praised the infirmary after making a visit of inspection, and by 1791 the number of beds had risen from twenty-seven to ninety-nine. Early Samaritan work took the form of providing wooden legs to all needy patients on their discharge, and half-a-dozen stools were given for lame patients "to rest their legs upon during their cure." About 1772 the board ordered a pair of clean sheets to be supplied every three weeks to the patients, an arrangement which the matron's discretion was later permitted to amplify.

### Sir Gilbert Scott's Building.

In 1817 a parcel of land on the south front was presented to the infirmary, but by 1859, with the rapid growth of the town, there was again congestion within, and pressing demands for further accommodation. The result was the employment of Sir Gilbert Scott, aided by Dr. Chadwick and Sir Douglas Galton, and the building of the existing infirmary. In this the corridor plan was given up in favour of the pavilion principle, which had been adopted at the Herbert Hospital, Woolwich, and the Hôpital Lariboisière in Paris. But the pavilion principle and the Gothic style are a combination more remarkable for the memories they excite than their practical advantages. In June 1869 patients were formally admitted. Two years later came the legacy of £30,000 from the Rev. Richard Brooke. But it was not till ten years had passed that the necessity for giving proper separate quarters to nurses was seriously admitted, when the first self-contained nurses' home was built. To the stimulus and foresight of the great former chairman, Mr. R. Benson Jowitt, the nurses also owe the Jowitt Pension Fund, christened after him by a circle of admirers who subscribed some £2,000 for this purpose. Mr. Jowitt was succeeded by Mr. Charles Lupton, whose varied services for the General Infirmary have so often filled the columns of The Hospital.

As to the Weatherill bequest, which fell due on February 9, 1905, "The Historical Sketch" somewhat cryptically remarks that the whole estate, estimated to be £117,000, "was to be given to the infirmary."

### The Reconstruction Scheme.

With the death of King Edward VII. in 1910 the Memorial Fund associated with his name was started, and in 1914 £111,000 had been raised, out of the £150,000 required for remodelling the Leeds General Infirmary. By this time, as our readers know, the scheme for five new theatres, a reconstructed out-patient department with special accommodation for casualty, nose, ear and throat, and orthopædic cases, together with wards for 100 additional beds, is nearly complete. The scheme also includes extensions to the pathological department, the nurses' home, and a new boiler-house and laundry. We hope to publish the plans and a description of the new buildings in an early issue.

On this note of expansion the sketch comes to an end. It should be studied in connection with the pamphlet issued by the infirmary in 1911, and the author's identity can be guessed between the lines. Of the lessons which it contains we will mention one only. It is that when the Eye and Ear Infirmary amalgamated with the greater institution the transfer increased the subscriptions to the combined institution. The problem of reconstructing old buildings so as to fit them for the latest demands of science and numbers is always complex. The Leeds Infirmary is an extreme example which Gilbert Scott architecture, site, and position in the city have all helped to complicate. What may be called the question of reconstruction as exemplified at Leeds General Infirmary might well form a special study which would place students and architects under a great debt.

After the war, audacity will be a word in frequent use in regard to many policies and projects affecting municipalities, public buildings, the protection and uplifting of men and women, and generally. Mr. Charles Lupton recognised the value of this word and adopted it under rather discouraging circumstances when launching a complete and adequate scheme for the extension of the Leeds Infirmary. It has not been found possible to clear all the site until after the war, but when this is done the boldness of Mr. Lupton's policy will be recognised as abundantly justified by its results.

